# Prediction of Antimicrobial and Antioxidant Activities of Mexican Propolis by ^1^H-NMR Spectroscopy and Chemometrics Data Analysis

**DOI:** 10.3390/molecules22071184

**Published:** 2017-07-14

**Authors:** J. Fausto Rivero-Cruz, Eduardo Rodríguez de San Miguel, Sergio Robles-Obregón, Circe C. Hernández-Espino, Blanca E. Rivero-Cruz, José Pedraza-Chaverri, Nuria Esturau-Escofet

**Affiliations:** 1 Facultad de Química, Universidad Nacional Autónoma de México, Ciudad Universitaria, 04510 Cd. Mexico, Mexico; joserc@unam.mx (J.F.R.-C.); erdsmg@unam.mx (E.R.d.S.M.); srobleso@hotmail.com (S.R.-O.); blancariv@unam.mx (B.E.R.-C.); pedraza@unam.mx (J.P.-C.); 2 Instituto de Química, Universidad Nacional Autónoma de México, Ciudad Universitaria, 04510 Cd. Mexico, Mexico; chernandeze@iquimica.unam.mx

**Keywords:** propolis extract, proton nuclear magnetic resonance, chemometrics, antibacterial, antioxidant capacity, multivariate statistical analysis

## Abstract

A feasibility study to predict antimicrobial and antioxidant activity properties of propolis extracts using 700-MHz ^1^H-NMR spectra and multivariate regression data analysis is presented. The study was conducted with thirty-five propolis samples to develop a rapid and reliable method for the evaluation of their quality. The extracts have been evaluated by measuring phenolic and flavonoid contents; the antioxidant activity; and the antimicrobial activity. The obtained spectral data were submitted to multivariate calibration (partial least squares (PLS) and orthogonal partial least squares (OPLS)) to correlate the relative intensity and position of NMR resonance peaks with the metabolites contents and biological activities. The developed PLS and OPLS model were successfully applied to the determination of the target properties for proof of the concept. The OPLS observed vs. predicted properties plots indicate the absence of systematic errors with determination coefficients between the ranges 0.7207 to 0.9990. Up to 86.1% of explication of variation in the spectral data and 99.9% in the measured properties were attained with 88.6% of prediction capabilities in the best case (*S. mutans* activity) according to the cross-validation procedure. The figures of merit of the developed PLS and OPLS methods were evaluated and compared as well.

## 1. Introduction

Propolis (bee glue), is a sticky dark-colored hive product collected by bees from living plant sources [1,2]. It possesses pharmacological activities such as antibacterial, antifungal, antioxidant, antitumoral, anti-inflammatory properties and is used extensively as an ingredient of candies, honeys, biopharmaceuticals, cosmetics and in beverages in various parts of the world where it is claimed to improve human health and to prevent diseases such as diabetes and cancer [3,4]. Recently, propolis has been proposed as chemical preservative in ground meat and as a germicide and insecticide for food packaging [4].

More than 300 compounds have been identified in different propolis samples [5]. This complex mixture contains a variety of chemical compounds such as flavonoid aglycones, phenolic acids and their esters, phenolic aldehydes, alcohols, ketones, sesquiterpenes, coumarins, steroids, amino acids and inorganic compounds [4,6,7,8]. The results have revealed that the propolis composition varies with geography and is strongly related with the flora surrounding the hive [1,4].

The main constituents of propolis in North America are flavonoids and phenolic acid esters [9]. Limited research has been conducted on the chemical composition and pharmacological properties of Mexican propolis. A study conducted by Velazquez et al., [10], investigated the antibacterial and free-radical scavenging (FRS) activities of propolis collected from three different areas of Sonora (Mexico). Navarro-Navarro et al., [11] reported the anti-*Vibrio* activity of propolis collected from three different regions of Sonora. Valencia et al., [3], studied the seasonal effect on the chemical composition and biological activities (antiproliferative and antioxidant activities) of Sonoran propolis.

The biological effects of propolis can be associated with its antioxidant activity, and in the last few decades new analytical techniques have been proposed to determine its antioxidant activity [12,13]. They are based, for example, on the determination of total phenolic and flavonoid contents or the antioxidant activity/capacity assays: 1,1-diphenyl-2-picrylhydrazyl (DPPH), ferric reducing/antioxidant power (FRAP), and generation of the (2,2′-azinobis-(3-ethylbenzothiazoline-6-sulfonic acid] (ABTS)) radical cation [14]. It is known that “quantitative evaluation of antioxidant capacity” needs more than one single assay method. A range of analytical methods have also been used to profile propolis, including chromatography techniques, linked to spectroscopic detection, resulting in various modern hyphenated techniques, e.g., GC-MS and HPLC-MS [15].

As already mentioned, propolis consists of a wide range of organic compounds of varying polarity and the only technique that can simultaneously examine waxes, terpenoids and phenolics is Nuclear Magnetic Resonance (NMR) spectroscopy [16]. One of the main advantages of this technique is that structural and quantitative information can be obtained for a wide range of chemical species in a single NMR experiment. NMR is frequently applied to samples that can be directly examined as liquids, but very simple extraction or sample preparation procedures may also be used [17,18].

Since the NMR pattern of natural products in propolis is extremely complex, the use of chemometric methods to analyze such complex spectral data sets is mandatory [19]. In the case of propolis, NMR with chemometric techniques have been proposed to identify and classify different propolis sources or geographic origins [18,20,21]. However, to the best of our knowledge, no study concerning the prediction of antioxidating and antibacterial properties of propolis based on multivariate calibration has been reported up to now.

In the present paper, the application of ^1^H-NMR coupled with multivariate statistical analysis, based on partial least squares, is employed to quantitatively predict the antibacterial and antioxidant activities of propolis extracts. The net analyte signal concept is used to determine the figures of merit of the developed methods. The study was conducted with 35 propolis samples obtained from different Mexican apiaries and four samples from out of the country (one from Ecuador and three from China) to develop a rapid and reliable method to evaluate the quality of them.

## 2. Results and Discussion

### 2.1. Extraction, Antioxidant and Antibacterial Activities

In this work, the ethanolic extracts of thirty-five samples of propolis obtained from different Mexican apiaries and four samples out of the country (one from Ecuador and three from China) were studied. The total phenolic and flavonoid contents were estimated using standard chemical assay procedures (Folin-Ciocalteu and AlCl_3_ methods). Several biological activities were evaluated including antioxidant capacity using the free radical scavenging DPPH assay and antimicrobial properties using *Streptococcus mutans*, *Streptococcus oralis* and *Streptococcus sanguinis* as test models. The results of the bioassays of the ethanolic extracts of propolis (EEP) samples are reported in Table 1. The total phenolic and flavonoid contents and antioxidant activity are in agreement with the literature for poplar propolis [3,6,10].

### 2.2. ^1^H-NMR

**Figure 1:**
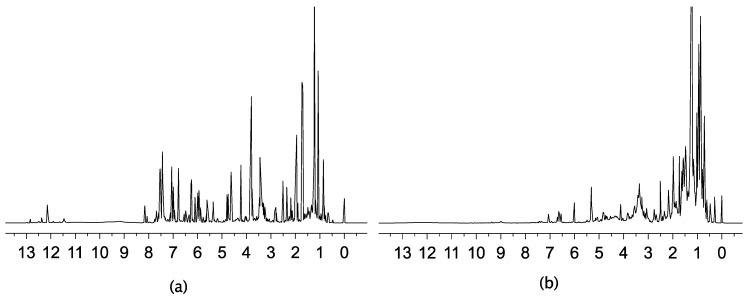
The 700-MHz ^1^H-NMR spectra of 40 mg of propolis sample dissolved in 0.6 mL of DMSO-*d*_6_ containing TMS. (**a**) Sample Puebla; Valsequillo 1 and (**b**) sample Puebla, Cuetzalan.

The ^1^H-NMR spectra of the EEP were recorded and, as an example, two selected spectra are shown in Figure 1. While spectrum Figure 1a belongs to an active extract, the Figure 1b one corresponds to an inactive one. In spectrum Figure 1a flavonoid compound signals are observed. Antioxidant and antimicrobial activities are well documented for this type of natural products [22,23]. The singlets around δ 12.0 ppm could be attributed to intramolecular hydrogen bond forming -OH groups frequently present in the A-ring of flavonoids. The aromatic protons of these phenolic constituents are observed between δ 6.0 ppm and 8.0 ppm. The signals between δ 6.0 ppm and 5.0 ppm could correspond to the vinylic protons of the C-ring of flavones present in the extract. The protons of the ABX system of the C-ring of a flavanone are expected between δ 5.0 ppm and 2.5 ppm. The singlet nearby δ 4.0 ppm could be attributed to a methyl moiety of an aromatic methoxy group frequently observed in flavonoids. On the other hand, spectrum Figure 1b is dominated by signals in the δ 2.0 ppm–0.5 ppm region, which could be originated from protons belonging to waxes or linear fatty acids whose contribution to antimicrobial or antioxidant activities may be considered less relevant.

### 2.3. Multivariate Analysis

The obtained spectral data were submitted to multivariate analysis; first, to study the variations among the sample spectra, and second, to correlate the relative intensity and position of NMR resonance peaks to antioxidant activity determined by DPPH, the total phenolic and flavonoid contents, and the antimicrobial activity.

**Figure 2:**
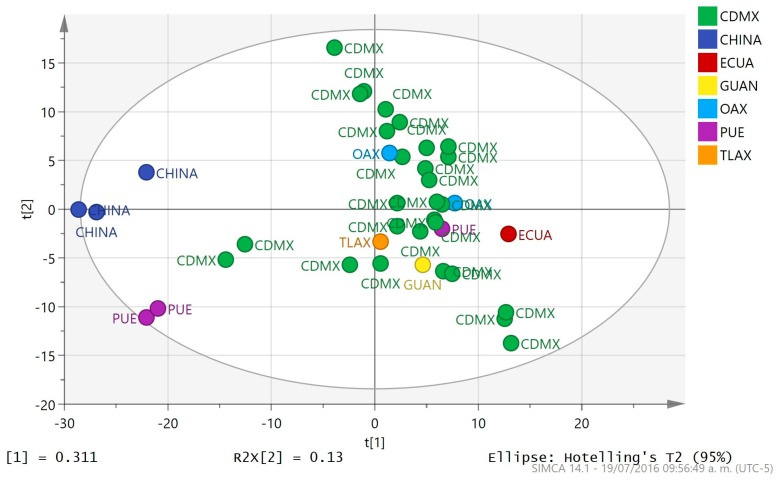
t2 vs. t1 score plot for PCA analysis of ^1^H-NMR spectra of propolis samples labeled according to their origin: Mexico City (CDMX), Puebla (PUE), Oaxaca (OAX), Guanajuato (GUAN), Tlaxcala (TLAX), Ecuador (ECUA) and China (CHINA).

Principal component analysis (PCA) is a technique used to emphasize variation and bring out strong patterns in a dataset. It’s often used to make data easy to explore and visualize. By examining the underlying structure of the variables, a new coordinate system is defined. The original variables are linear combined in new ones, named principal components, and in such form the dimensionality, i.e., complexity of the data space is reduced. The PCA analysis of the ^1^H-NMR spectra of propolis showed that with six components 73.3% of spectral variation was explained (*R2X*(cum)). This value is a measure of the amount of information contain within the model to explain the dispersion observed when comparing the different sample spectra. The percent of variation that can be predicted by the model according to a leave-one-out cross-validation procedure reached 48.7% (*Q2X*(cum)). Cross-validation is used to estimate how accurately a predictive model will perform in practice and it is employed as an estimator of the prediction behavior in the absence of an independent set of samples for validation. A quick view of the sample distribution according to spectral similarities in the plot of scores t2 vs. t1 (Figure 2), where the scores are the values of the new variables, indicated a natural tendency of the samples of the same apiary or apiaries to lie in close proximity, but no grouping among the samples according to their different origins is in fact observed in the plot. This was confirmed by the tolerance ellipse that defines a 95% confidence interval for a Hotelling *T*^2^ test, indicating that all samples can be considered as representative of the same population. It was also observed that although some samples lay very close to the limits of the ellipse, no outliers were really present in the data. On the basis of the analysis of the loadings plots (supplementary information), the differences among samples are mainly of quantitative rather than of qualitative nature, as the chemical shifts in their NMR spectra cannot be assigned to any particular discriminant unique features. In addition, the analysis showed that some samples had distance to the model (DModX) values just slightly above the critical value; however, it was decided to include them in further treatments. DModX is the distance of an observation in the data set to the X model plane or hyperplane, which is proportional to the residual standard deviation (RSD) of the X observation. Interestingly such values corresponded to samples outside Mexico City (Puebla) and even the country (China).

The spectra were also treated with PLS regression analysis. PLS is a method for relating two data matrices, X (the ^1^H-NMR spectra) and Y (the properties, e.g., phenol content), by a linear multivariate model, but goes beyond traditional regression in that it models the structure of X and Y by PCA analysis as well. The regression is then performed with the analogous of the principal components, named latent variables, of the X and Y matrices.

In a first step the complete spectral range was employed (0.5 ppm–13.5 ppm). However, from the analysis of the regression coefficients, an improvement in regression parameters was observed when the range was restricted to 0.5 ppm–8.2 ppm, and further processing was done using this interval.

In Table 2, the values of *R2X* (cum), *R2Y* (cum), and *Q2X* (cum) for the different evaluated properties are indicated. *R2Y* (cum) has the same meaning that *R2X* (cum) but instead of analyzing spectrum data, it considers the data contained in Y matrix (responses). Values for the determination coefficient (*R*^2^), the Root Mean Square Error of Estimation (RMSEE) and the Root Mean Standard Error of Cross Validation (RMSECV), as well as for the number of latent variables used in the models are in addition included. RMSEE and RMSECV are descriptive statistic parameters that allow the accuracy of the model to be quantitatively measured. The numbers of significant latent variables were selected according to the cross-validation rules included in SIMCA for such purposes: (i) Q^2^ > limit, where limit = 0 for PLS models with more than 100 observations. Limit = 0.05 for PLS models with 100 observations or less, and limit = 0.01 for OPLS; (ii) Q^2^V > limit for at least 20% of the *y*-variables when M ≥ 25 or sqrt(M) when M < 25, where M = number of *y*-variables and Q^2^V is Q^2^ for individual variables. Overall, good performance is achieved for all properties and no systematic variations are detected based on the slope and intercept values of the regression equations between the defined and predicted values.

With the aim of improving the prediction error for the data by eliminating orthogonal variation in X, the OPLS method was tested. This orthogonal variation is due to sources of variation which are not correlated with the measured properties, i.e., it is the non-predictive part of the variation in the X matrix. As observed in the same Table, in general, better performance characteristics are obtained, i.e., a reduction in RMSEE and RMSECV, an increase in *R2X* (cum), *R2Y* (cum), *Q2* (cum) and *R*^2^, without deterioration in the regression equations, as most of the points fall close to the 45 degree line, with no systematic errors present. Values of *R*^2^ ranging from 0.7207 to 0.9990 were observed for the regression lines indicating strong relationships between the defined and predicted values of total phenol and flavonoid content, DPPH radical scavenging activity, and in vitro antibacterial activity against *Streptococcus mutans*, *Streptococcus oralis* and *Streptococcus sanguinis.* At this point it is also important to mention that the residual plots of the data for both the PLS and OPLS analyses showed no systematic trends and a satisfactory fit to normal probability plots, thus confirming the correct application of the models.

**Figure 3:**
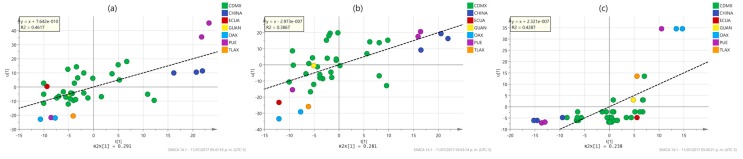
Inner relationship plots for the X-scores (u-data) and Y-scores (t data) between the first latent variables of the PLS models for (**a**) flavonoid content; (**b**) antioxidant (DPPH) content; and (**c**) antimicrobial *S. mutants* MIC activity, labeled according to their origin: Mexico City (CDMX), Puebla (PUE), Oaxaca (OAX), Guanajuato (GUAN), Tlaxcala (TLAX), Ecuador (ECUA) and China (CHINA). Inside the figures the lineal regression equation and determination coefficient value of the data are reported.

**Figure 4:**
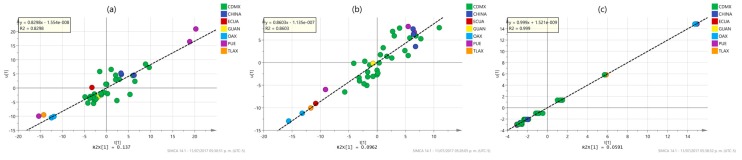
Inner relationship plots for the X-scores (u-data) and Y-scores (t data) between the first latent variables of the OPLS models for (**a**) flavonoid content; (**b**) antioxidant (DPPH) content; and (**c**) antimicrobial *S. mutants* MIC activity, labeled according to their origin: Mexico City (CDMX), Puebla (PUE), Oaxaca (OAX), Guanajuato (GUAN), Tlaxcala (TLAX), Ecuador (ECUA) and China (CHINA). Inside the figures the lineal regression equation and determination coefficient value of the data are reported.

To better understand the differences between PLS and OPLS methods to model and predict the response values, some characteristic examples of the inner relationship plot of the analysis of the models described in Table 2 are shown in Figure 3 and Figure 4. These plots represent the correlation between the scores of the predictors (u data) and response (t data). A perfect match between the X- and the Y-data is observed when all data points are located on the diagonal line with slope equal to one. Conversely, when there is a weak correlation structure between X and Y, there is a considerable spread of the points around such line. The plot is also useful to identify curved (non-linear) relationships between the predictors and the responses and to identify outliers in X- and Y-data, and in the relationship between X and Y. As observed in Figure 3, PLS models give moderate correlations between spectra and properties, denoted by both medium values of the determination coefficient (*r*^2^ values ranging from 0.3867 to 0.4617) and significant spread of the samples along the reference line. Some samples inside Mexico City (CDMX) and outside the city (Oaxaca (OAX), Puebla (PUE) and Tlaxcala (TLAX)) and even the country (China) look like outliers in the relationship between the X- and Y-blocks. In contrast, OPLS modeling produces very strong correlation results (Figure 4) as high reduction in the spread of the samples along the reference line is observed with a considerable increase in the values of the determination coefficients (*r*^2^ values ranging from 0.8298 to 0.999). This time, the outlier samples observed in PLS modeling practically disappear at all, suggesting that OPLS modeling reduces a particular source of variability in the NMR chemical shifts associated with such samples. Further analysis of the regression coefficients of the PLS and OPLS models will be latter performed to identify the chemical shifts responsible for differences in PLS and OPLS modeling. Similar results were observed for the properties not shown in Figure 3 and Figure 4.

**Figure 5:**
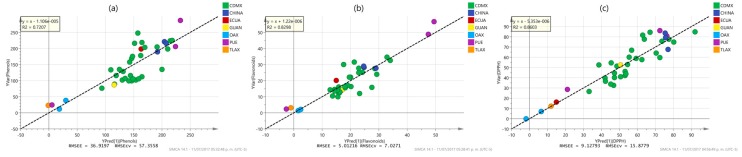
OPLS model observed vs. predicted (**a**) phenol content; (**b**) flavonoid and (**c**) antioxidant (DPPH) content plot of propolis samples labeled according to their origin: Mexico City (CDMX), Puebla (PUE), Oaxaca (OAX), Guanajuato (GUAN), Tlaxcala (TLAX), Ecuador (ECUA) and China (CHINA).

**Figure 6:**
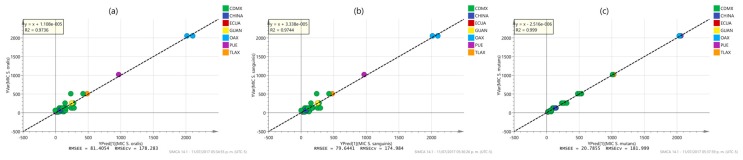
OPLS model observed vs. predicted antimicrobial MIC activity (**a**) *S. oralis*; (**b**) *S. sanguinis*, and (**c**) *S. mutants*, plot of propolis samples labeled according to their origin: Mexico City (CDMX), Puebla (PUE), Oaxaca (OAX), Guanajuato (GUAN), Tlaxcala (TLAX), Ecuador (ECUA) and China (CHINA).

In Figure 5 and Figure 6 the observed vs. predicted values plots of the different properties using OPLS modeling are shown. It is obvious that the samples are not homogeneously distributed, as most of the observations are clustered and others grouped outside the main array. This is especially true concerning the antibacterial activity, in which it is clearly noted that the inclusion of samples outside Mexico City (CDMX), especially Puebla (PUE), Oaxaca (OAX) and Tlaxcala (TLAX), allows a more suitable prediction due to the extend range that such samples confers for modeling. This fact is reflected in the RMSEE and RMSECV values which are lower for phenol and flavonoids contents and DPPH activity than for MIC assays. The plots also shown that although phenol and flavonoid contents as well as DPPH activity are almost equally spaced between samples, the MIC activities are not. This trend clearly indicates that although the compounds that produce the antioxidant properties are presents in an extended range of concentrations in the samples discernable by the measuring method by a continuous variable, not all of them have antimicrobial activities. In addition, the observed grouping in the MIC activities is a logical consequence of the nature of the MIC analysis (two-fold serial dilutions) which produces a discrete variable as results and the similarities between samples concerning this parameter. The low antibacterial activity of certain samples, especially those from Puebla (PUE), Oaxaca (OAX) and Tlaxcala (TLAX), is clearly related to their low phenol and flavonoid contents, as expected for the antioxidant capacity of such compounds. The inclusion of new samples with a diversity of origins and further characterization of the propolis samples will be a recommendable form to extend the model prediction capabilities.

**Figure 7:**
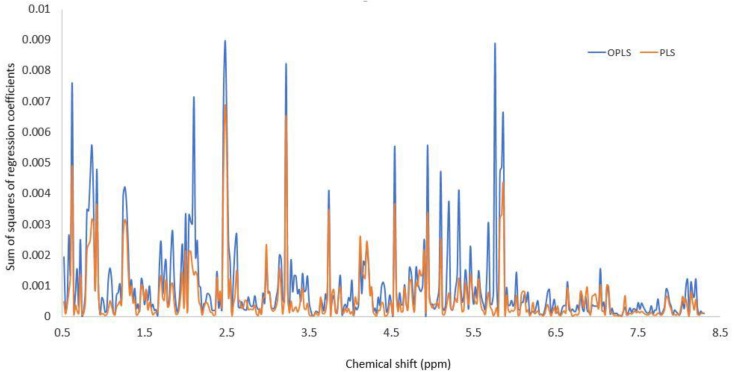
Sum of squares of the regression coefficient vectors of the PLS and OPLS models for all properties.

In Table 3 the figures of merit of the PLS and OPLS methods are reported. As observed, both methods perform similarly. Clearly the orthogonal signal correction of OPLS algorithm filters uncorrelated variability in the sample spectra, thus increasing the selectivities up to its maximum values of 1.00, thus allowing better prediction capabilities of the model as measured by *Q2X* (cum). By comparing PLS and OPLS selectivity results, this uncorrelated variability has an average value of 17%. A comparison of the sum of squares of the regression coefficients for all properties for the PLS and OPLS models (Figure 7) reveals that both models give high importance to predict the target properties to the 0.5 ppm–6.0 ppm region; however the OPLS technique give more relevance to the 1.7 ppm–2.2 ppm and 5 ppm–5.8 ppm regions of the ^1^H-NMR spectra, which according to the discussion above, such chemical shifts were mainly attributed to protons belonging to waxes or linear fatty acids and to the vinylic protons of the C-ring of flavones present in the extract, respectively, which content seems to be determinant in the values of the of total phenol and flavonoid content, DPPH radical scavenging activity, and in vitro antibacterial activity against *Streptococcus mutans*, *Streptococcus oralis* and *Streptococcus sanguinis*.

Further improvement in the developed methods may be performed for the implementation of potential quality control protocols and more accurate predictions by the inclusion of new samples with a diversity of origins, the determination of flavanones and dihydroflavonols with specific methods and the addition of IC50 values of the samples as a target property. Specifically, as the method which involves the measurement at 410 nm–430 nm after addition of AlCl_3_ solution is selective only for flavonols (quercetin, morin, kaempferol and rutin) and flavones luteolin, complementing the data with a measurement procedure at 510 nm in the presence of NaNO_2_ in alkaline medium, may be a feasible form to evaluate rutin, luteolin and catechins, although it should be considered that phenolic acids exhibit considerable absorbance at this wavelength. With this new information, an improved interpretation of the relationship between polyphenols/flavonoids quantification and antimicrobial activity may be anticipated. This article allows a proof of the concept for such purposes.

## 3. Materials and Methods

### 3.1. Samples

Thirty-nine propolis samples were provided by local beekeepers (Federico Palma Valderrama and MVZ Ángel López Ramírez). The propolis samples were collected between 2011 and 2014 (Table 1). These 39 samples were obtained by different harvesting methods, 18 by scraping, one by wooden wedges (3 mm−5 mm thick), and 16 by plastic nets (mesh size = 2 mm).

### 3.2. Chemicals and Reagents

The reagents 6-hydroxy-2,5,7,8-tetramethylchroman-2-carboxylic acid (Trolox, 97%), gallic acid, 2,2-diphenyl-1-picrylhydrazyl (DPPH), sodium carbonate, chlorhexidine gluconate, and quercetin, were supplied by Sigma-Aldrich (St. Louis, MO, USA). Ethanol was supplied by Merck (Darmstadt, Germany). Dimethyl sulfoxide-*d*_6_ (D, 99.9%) +0.05% *V*/*V* TMS was supplied by Cambridge Isotope Laboratoriesn (Tewksbury, MA, USA).

### 3.3. Extract Preparation

Five g of each crude propolis sample was extracted with ethanol (250 mL) at room temperature during 7 days. Each extract was taken to dryness under reduced pressure to afford the ethanolic extracts of propolis (EEP). Extracts were stored at −20 °C until analysis.

### 3.4. DPPH Radical Scavenging Assay

DPPH radical scavenging activity was investigated according to the method of Cheng et al. [24]. Briefly, an ethanolic solution of DPPH (0.208 mM, 0.1 mL) was mixed with extract (1 mg/mL, 0.1 mL) or Trolox (positive control, 1 mg/mL). The 96-well plate was incubated in the dark at room temperature for 20 min, and the absorbance was recorded at 540 nm. The percentage inhibition of the DPPH by each sample was calculated considering the percentage of the steady DPPH in solution after the reaction. All the determinations were performed in triplicates. The percentage scavenging effect was calculated as:

Scavenging rate = [1 − (A_2_ − A_1_)/A_0_] × 100%

where A_0_ is the absorbance of the control, A_1_ the absorbance in presence of the sample, A_2_ the absorbance of sample without DPPH radical.

### 3.5. Total Phenolic Content

The total phenolic content of propolis was determined as described by Singleton and Rossi [25] and Popova et al. [26]. Briefly, propolis extract (1 mg/mL, 20 µL) and Folin-Ciolcateau reagent (80 µL) were mixed well during 5 min and 7.5% sodium carbonate solution (80 µL) was added. The plate was covered and incubated in the dark (at room temperature) during 2 h. The absorbance was measured at 760 nm with a spectrophotometric microplate reader (Benchmark 11130, Bio-Rad, Hercules, CA, USA). Distilled water was used as a blank. The obtained absorbances were interpolated in a calibration curve (y = 4.10x + 0.0324, *R*^2^ = 0.9980) of gallic acid. The results were expressed as mg equivalents of gallic acid/g of dry extract of propolis (EEP). All the determinations were performed in triplicates. The total phenolic content was estimated using gallic acid and quercetin as standards.

### 3.6. Total Flavonoid Content

The concentration of flavonoids was determined using the method described by Marquele et al. [27] using aluminum chloride reagent (2% in methanol). Extract (100 µL) was mixed with aluminum chloride solution (2% in methanol, 100 µL). After incubation for 30 min at room temperature, the absorbance was read at 420 nm and concentrations of flavonoids were determined from a calibration curve obtained with quercetin. The obtained absorbances were interpolated in a calibration curve (y = 16.33x + 0.1032, *R*^2^ = 0.9993) of quercetin. The results were expressed as mg equivalents of quercetin/g of dry extract of propolis (EEP).

### 3.7. Determination of Minimum Inhibitory Concentration (MIC)

The in vitro antibacterial activity of each EEP was determined using a broth microdilution test as recommended by Clinical and Laboratory Standards Institute M7-A4 for bacteria CLSI [28]. The MIC was defined as the lowest concentration of the test agent that had restricted growth to a level <0.05 at 660 nm after incubation at 37 °C for 16 h–24 h. Growth inhibitory effects of the extracts were tested against *Streptococcus mutans* (ATCC 10449), *Streptococcus oralis* (ATCC 35037) and *Streptococcus sanguinis* (ATCC 10556). The procedures employed were as described previously [29]. Sterile 96-well microtiter plates were used. Each well in the microtiter plate contained *Streptococcus* (final concentration of 5 × 10^5^ colony forming units (CFU)/mL), serially diluted EEP, and the appropriate growth medium. Triplicate samples were performed for each test concentration. The controls included inoculated growth medium without test compounds. Sample blanks contained uninoculated growth medium only. All plates were incubated at 37 °C under appropriate atmospheric conditions with growth estimated spectrophotometrically (A_660_ nm) after 24 h using a microtiter plate reader. The MIC value for each test organism was defined as the minimum concentration of test compound limiting turbidity to <0.05 A_660_ nm. As a positive control, chlorhexidine gluconate (CHX) was used.

### 3.8. NMR Experiments

All ^1^H-NMR spectra of propolis extract were collected at 300 K on an Avance III HD 700 MHz spectrometer (Bruker, Billerica, MA, USA) equipped with a 5-mm z-axis gradient inverse probe. The spectrum was recorded using the standard single-pulse sequence, with the 90° pulse length of 7.76 µs. 128 scans were collected into 32 k data points using a spectral width of 14 kHz with a relaxation delay of 5 s, and acquisition time 2.3 s. The free induction decays (FIDs) were multiplied by an exponential function with a line-broadening factor of 0.3 Hz before Fourier transformation. The ^1^H-NMR spectra were manually corrected for phase and baseline distortion using MestReNova software (version 10.0.2, Mestrelab Research, Santiago de Compostela, Spain). The ^1^H-NMR chemical shifts were referenced to TMS signal at 0.0 ppm. 20 mg of sample was weighed out and dissolved in 0.5 mL of DMSO-*d*_6_ containing 0.03% TMS.

### 3.9. Data Processing for Multivariate Analysis

Using the software MestReNova each one-dimensional ^1^H-NMR spectrum was sliced into 0.02 ppm sections between 0.5 ppm and 13.5 ppm. Processed spectra were normalized to the total average sum of integrals. The resulting normalized integrals composed the data matrix that was submitted to multivariate analysis.

### 3.10. Multivariate Analysis

Principal component analysis (PCA), an unsupervised explorative data analysis technique, and partial least squares regression projection to latent structures (PLS), and its orthogonal form (OPLS), regression models employed to find the fundamental relations between two data matrices, were used for data analysis. The quality of the models was evaluated based on the diagnostic tools: the cumulative modeled variation in matrix X, *R2X* (cum), the proportion of the variance of the response variable that is explained by the model, *R2Y* (cum), and the predictive ability parameter, *Q2* (cum).

All statistical data analyses were performed as implemented in the SIMCA 14.1.0.2047 software (MKS Umetrics, Malmö, Sweden) using unit variance (UV) scaling after optimization of this variable. For figures of merit determination an in house-made MATHLAB program was used with the outputs of the SIMCA software.

### 3.11. Figures of Merit

A figure of merit is a quantity used to characterize the performance of an analytical method. Well known in univariate calibration (where a single number is measured for each sample), the figures of merit can also be defined in multivariate calibration in an easy form through the Net Analyte Signal (NAS) concept [30,31,32].

The NAS concept arises from the fact that a prediction sample spectrum may have varying contributions from other sample components. Hence, the spectrum can be decomposed in two orthogonal parts: a part that can be uniquely assigned to the analyte of interest (the NAS), and the remaining part that contains the contribution from other components. Using the NAS, a multivariate calibration model can be represented in a pseudo-univariate plot. NAS is evaluated as:

$$NAS_{i}=(x_{i}\cdot b)\cdot {\left(b^{T}\cdot b\right)}^{-1}\cdot b^{T}$$

where *x_i_* is a sample spectrum after preprocessing and b is a column vector of the PLS regression coefficients.

*Accuracy*. It expresses the proximity between the reference value and that predicted by the model. It can be measured in many forms, among them the Root Mean Square Error of Estimation (RMSEE) and the Root Mean Standard Error of Cross Validation (RMSECV):

$$RMSEE=\sqrt{\frac{\sum_{i=1}^{n}{\left(y_{i}-{\hat{y}}_{i}\right)}^{2}}{n-1}}$$

where $y_{i}$ *y* ${\hat{y}}_{i}$ are the estimated and reference values, respectively, of the *i*, simple and *n* the total number of samples. RMSECV is calculated in a similar way by leaving out all permutations of a given number of samples from the training set and computing the total RMSEE value of the procedure by adding the RMSEE value for each calibration. RMSEE measures the fit of the model while RMSECV its predictive power.

*Selectivity (sel)*. It expresses the fraction of the signal that changes when the concentration of the analyte varies in one unit. It can be evaluated through the NAS concept as:

$$sel=\frac{{||s}_{k}^{*}||}{{||s}_{k}||}$$

where ${||s}_{k}||$ stands for the norm of the sensitivity coefficients of the spectra containing the analyte *k* at unit concentration and ${||s}_{k}^{*}||$ for that corresponding to its NAS.

*Sensitivity (sen)*. It is a measure of the response change with analyte concentration. In multivariate context represents the NAS generated by an analyte concentration equal to unity, and is evaluated through:

$$sen={||s}_{k}^{*}||=\frac{1}{\left||b|\right|}$$

where ||b|| is the norm of the vector of regression coefficients of the calibration model.

*Analytical sensitivity (γ)*. Defined by the ratio between sensitivity and instrumental noise, δx, as:

$$\gamma =\frac{sen}{\left|\delta x\right|}$$

it allows a comparison between methodologies based on very different instrumental measurements, as it is independent on the measured signal. The inverse of this parameter, γ^−1^, establishes a minimum concentration difference that is discernible by the analytical method considering the random experimental noise as the only source of error.

*Limit of detection (LD)*. It is defined as the minimum detectable value of the net signal (or concentration) for which the probabilities of false negatives (β) and false positives (α) are at maximum 5%. It is evaluated as:

$$LD=3.3\delta x\frac{1}{sen}$$

*Limit of* *quantitation (LQ)*. It determines the net signal or analyte concentration value which can be estimated with a relative error lower than 10%. It is evaluated as:

$$LQ=10\delta x\frac{1}{sen}$$

## 4. Conclusions

The total phenol and flavonoid contents as well as the antioxidant (DPPH) and in vitro antibacterial activities against *Streptococcus mutans*, *Streptococcus oralis* and *Streptococcus sanguinis* were quantitatively correlated with ^1^H-NMR spectra data using PLS and OPLS calibration models. Preliminary PCA analysis was performed to characterize the samples and to identify possible outliers. Results indicated a natural tendency of the samples of the same apiary or apiaries to lie in close proximity. PLS and OPLS regression methods gave excellent calibration models, although OPLS performed better in terms or the RMSEE, RMSECV, *R2X* (cum), *R2Y* (cum), *Q2* (cum) and *R*^2^ values, as expected due to the separation of the systematic variation in the predictive and non-predictive parts. The figures of merit of the developed methods were determined as well, so that methods were characterized in terms of their limits of detection and quantitation, sensitivity, selectivity and analytical sensitivity values (Table 3). The inclusion of new samples with a diversity of origins will be a recommendable form to improve the prediction capabilities of the developed models. The study demonstrates for the first time the possibility to develop a rapid and reliable method based on ^1^H NMR for the evaluation of the quality of propolis samples of different origin in terms of the evaluation of their chemical composition and antioxidant and antibacterial properties.

## Figures and Tables

**Table 1:** Characteristics of the of EEP samples from Mexico City (CDMX), Puebla (PUE), Oaxaca (OAX), Guanajuato (GUAN), Tlaxcala (TLAX), Ecuador (ECUA) and China (CHINA) used in this study.

Source of Sample	Date of Harvesting	Harvesting Method	Total Phenols ^a^	Total Flavonoids ^b^	% DPPH ^c^	MIC (µg/mL)		
						*S. oralis*	*S. sanguinis*	*S. mutans*
**Mexico City (CDMX)**								
***Tlalpan***								
Topilejo 1	September 2011	plastic nets	112.7	15.7	42.3	128	128	256
Topilejo 2	October 2011	plastic nets	116	24.9	46.1	128	128	256
Topilejo 3	November 2011	plastic nets	106.7	12.6	40.2	32	32	64
Topilejo 4	October 2012	plastic nets	134.1	14.2	52.5	128	128	256
Topilejo 5	October 2013	plastic nets	101.3	13.6	40.8	128	128	256
Topilejo 6	October 2014	plastic nets	106.3	12.1	32.6	128	128	128
***Xochimilco***								
San Luis Tlaxialtemalco 1	October 2011	scraping	99.7	22.1	66.8	512	512	512
San Luis Tlaxialtemalco 2	November 2011	scraping	157.3	15.6	52.9	128	128	256
San Luis Tlaxialtemalco 3	October 2012	scraping	76.4	14.1	26.6	512	512	1024
San Luis Tlaxialtemalco 4	October 2013	scraping	175.9	17.1	60.2	64	128	128
San Luis Tlaxialtemalco 5	October 2013	plastic nets	116.4	14.5	54.6	128	128	256
San Luis Tlaxialtemalco 6	November 2013	plastic nets	134.6	13.5	44.6	128	128	256
***Milpa Alta***								
San Pablo Oztotepec 1	October 2011	plastic nets	101.3	25.2	59.6	128	128	128
San Pablo Oztotepec 2	October 2011	scraping	247.3	27.8	84.4	32	32	32
San Pablo Oztotepec 3	November 2012	plastic nets	128.9	12.2	39.6	128	128	256
San Pablo Oztotepec 4	October 2011	scraping	223.2	34.6	77.5	32	32	64
San Pablo Oztotepec 5	November 2012	scraping	225.4	32.6	75.6	32	32	64
San Pablo Oztotepec 6	October 2011	plastic nets	135.2	27.5	64	64	64	128
San Pablo Oztotepec 7	October 2012	scraping	203.8	31.5	81.7	16	16	32
San Pablo Oztotepec 8	October 2013	plastic nets	204.3	16.3	84.8	32	32	64
San Pablo Oztotepec 9	October 2012	scraping	218.9	22.1	77.5	32	32	32
San Pablo Oztotepec 10	November 2012	scraping	178.3	16.3	57.5	64	128	128
San Pablo Oztotepec 11	October 2013	scraping	168.6	16.1	58.8	64	64	128
San Antonio Tecomitl 1	November 2013	scraping	198.5	23.9	75	32	64	128
San Antonio Tecomitl 2	October 2011	scraping	215.7	30	51.2	32	64	64
San Antonio Tecomitl 3	October 2011	plastic nets	99.6	10.1	33.8	256	256	512
San Antonio Tecomitl 4	October 2013	plastic nets	105.3	13.1	43.6	64	64	128
San Antonio Tecomitl 5	October 2012	plastic nets	89.8	10.5	36.5	256	256	512
**Puebla (PUE)**								
Valsequillo 1	October 2011	wooden wedges	205.6	48.7	80.9	16	32	32
Valsequillo 2	October 2012	scraping	287.2	56.7	86	16	16	16
Cuetzalan	October 2011	scraping	24.7	2.3	28.7	1024	1024	2048
**Oaxaca (OAX)**								
Pinotepa Nacional 1	October 2011	scraping	38.5	2.1	7.1	2048	2048	2048
Pinotepa Nacional 2	October 2012	scraping	12	1.3	0	2048	2048	2048
**Guanajuato (GUAN)**								
Silao	October 2012	plastic nets	87.3	14.2	52.4	256	256	512
**Tlaxcala (TLAX)**								
Tlaxcala	October 2011	scraping	23.5	3.1	12.1	512	512	1024
**South America (ECUA)**								
Quito/Ecuador	2011		198.3	20.1	16.3	64	128	128
**China (CHINA)**								
China 1 *	2013		221.7	28.3	83.7	64	64	64
China 2 *	2013		215.6	29.1	79.1	64	64	64
China 3 *	2012		189.9	27.8	67.8	64	64	128
**Positive control ^†^**						0.12	0.12	0.24

^a^ Expressed in mg GAE/g EEP; ^b^ Expressed in mg QE/g EEP; ^c^ DPPH radical scavenging activity (percent); * Purchased in a local market in Mexico City; ^†^ Chlorhexidine gluconate.

**Table 2:** Results of the PLS and OPLS modeling of propolis samples.

Property	Number of Latent Variables	Regression Equation ^a^	*R*^2^	R2X (cum)	R2Y (cum)	Q2 (cum)	RMSEC	RMSECV
**PLS**								
Phenol content	2	*x* − 1.397 × 10^−6^	0.6003	0.400	0.600	0.263	43.548	56.086
Flavonoid content	2	*x* + 1.212 × 10^−7^	0.7204	0.397	0.720	0.482	6.334	8.305
DPPH	2	*x* + 1.61 × 10^−7^	0.7110	0.388	0.711	0.325	12.760	18.511
MIC (*S. oralis*)	3	*x* + 1.165 × 10^−5^	0.9411	0.460	0.941	0.778	118.005	244.768
MIC (*S. sanguinis*)	3	*x* + 7.404 × 10^−6^	0.9434	0.460	0.943	0.784	115.076	241.684
MIC (*S. mutants*)	3	*x* + 2.033 × 10^−6^	0.9245	0.463	0.925	0.696	156.141	322.235
**OPLS**								
Phenol content	1 + 2+ 0	*x* − 1.106 × 10^−5^	0.7207	0.472	0.721	0.249	36.920	57.356
Flavonoid content	1 + 2 + 0	*x* + 1.22 × 10^−6^	0.8298	0.463	0.830	0.627	5.012	7.027
DPPH	1 + 3 + 0	*x* − 5.353 × 10^−6^	0.8603	0.542	0.860	0.515	9.128	15.878
MIC (*S. oralis*)	1 + 4 + 0	*x* + 1.108 × 10^−5^	0.9736	0.596	0.974	0.850	81.405	178.283
MIC (*S. sanguinis*)	1 + 4 + 0	*x* + 3.338 × 10^−5^	0.9744	0.595	0.974	0.854	79.644	174.984
MIC (*S. mutants*)	1 + 12 + 0	*x* − 2516 × 10^−6^	0.9990	0.861	0.999	0.886	20.785	181.999

^a^ observed vs. predicted values.

**Table 3:** Figures of merit of the PLS and OPLS models.

Figure of Merit	Phenol Content	Flavonoid Content	DPPH	MIC (*S. oralis*)	MIC (*S. sanguinis*)	MIC (*S. mutants*)
**PLS**						
LD	0.37 mg GAE g EEP^−1^	0.35 mg QE g EEP^−1^	0.40%	0.51 µg mL^−1^	0.51 µg mL^−1^	0.53 µg mL^−1^
LC	1.12 mg GAE g EEP^−1^	1.08 mg QE g EEP^−1^	1.21%	1.55 µg mL^−1^	1.55 µg mL^−1^	1.61 µg mL^−1^
Evaluated linearity	12–287.20 mg GAE g EEP^−1^	1.30–56.70 mg QE g EEP^−1^	0.40–86%	16–2048 µg mL^−1^	16–2048 µg mL^−1^	16–2048 µg mL^−1^
Sens	39.93 g EEP mg GAE^−1^	41.39 g EEP mg QE^−1^	36.80%^−1^	25.23 mL µg^−1^	25.27 mL µg^−1^	25.89 mL µg^−1^
Sel	0.87	0.87	0.86	0.79	0.79	0.79
γ	8.94 g EEP mg GAE^−1^	9.28 g EEP mg QE^−1^	8.28%^−1^	6.45 mL µg^−1^	6.46 mL µg^−1^	6.19 mL µg^−1^
γ^−1^	0.11 mg GAE g EEP^−1^	0.11 mg QE g EEP^−1^	0.12%	0.15 µg mL^−1^	0.15 µg mL^−1^	0.16 µg mL^−1^
**OPLS**						
LD	0.45 mg GAE g EEP^−1^	0.39 mg QE g EEP^−1^	0.49%	0.67 µg mL^−1^	0.66 µg mL^−1^	0.66 µg mL^−1^
LC	1.38 mg GAE g EEP^−1^	1.20 mg QE g EEP^−1^	1.50%	2.03 µg mL^−1^	1.86 µg mL^−1^	1.99 µg mL^−1^
Evaluated linearity	12–287.20 mg GAE g EEP^−1^	1.30–56.70 mg QE g EEP^−1^	0.40–86%	16–2048 µg mL^−1^	16–2048 µg mL^−1^	16–2048 µg mL^−1^
Sens	40.34 g EEP mg GAE^−1^	45.12 g EEP mg QE^−1^	37.76%^−1^	28.92 mL µg^−1^	29.13 mL µg^−1^	29.59 mL µg^−1^
Sel	1.00	1.00	1.00	1.00	1.00	1.00
γ	7.24 g EEP mg GAE^−1^	8.35 g EEP mg QE^−1^	6.66%^−1^	4.92 mL µg^−1^	4.95 mL µg^−1^	5.03 mLµg^−1^
γ^−1^	0.14 mg GAE g EEP^−1^	0.12 mg QE g EEP^−1^	0.15%	0.20 µg mL^−1^	0.20 µg mL^−1^	0.20 µg mL^−1^

## Supplementary Materials

Supplementary Materials are available online.
