# Supplementary material for: Prediction of Antimicrobial and Antioxidant Activities of Mexican Propolis by 1H-NMR Spectroscopy and Chemometrics Data Analysis
Source: Molecules. 2017 Jul 14;22(7):1184. doi: 10.3390/molecules22071184 (PMC6152011; doi:10.3390/molecules22071184)
Supplement: Supplementary file 1 [file molecules-22-01184-s001.pdf]

molecules

Prediction of antimicrobial and antioxidant activities of Mexican propolis by ^{1}H NMR spectroscopy and chemometrics data analysis.

J. Fausto Rivero-Cruz [1], Eduardo Rodríguez de San Miguel [1], Sergio Robles-Obregón [1,¥] , Circe C. Hernández-Espino [2], Blanca E. Rivero-Cruz [1], José Pedraza-Chaverrí [1], Nuria Esturau-Escofet [2,*]

1 Facultad de Química, Universidad Nacional Autónoma de México, Ciudad Universitaria, 04510, Cd. México, México. joserc@unam.mx (J. F. R-C.), erdsmg@unam.mx (E. R. S. M.), srobleso@hotmail.com (S. R-O.), blancariv@unam.mx (B. E. R-C.), pedraza@unam.mx (J. P-C.).

2 Instituto de Química, Universidad Nacional Autónoma de México, Ciudad Universitaria, 04510, Cd. México, México. chernandeze@iquimica.unam.mx (C. C. H-E.); nesturau@iquimica.unam.mx (N. E-E.).

* Correspondence: nesturau@iquimica.unam.mx; Tel.: +52 (55) 56 22 47 70 ext 45648

Academic Editor: name

Received: date; Accepted: date; Published: date

molecules

MDPI

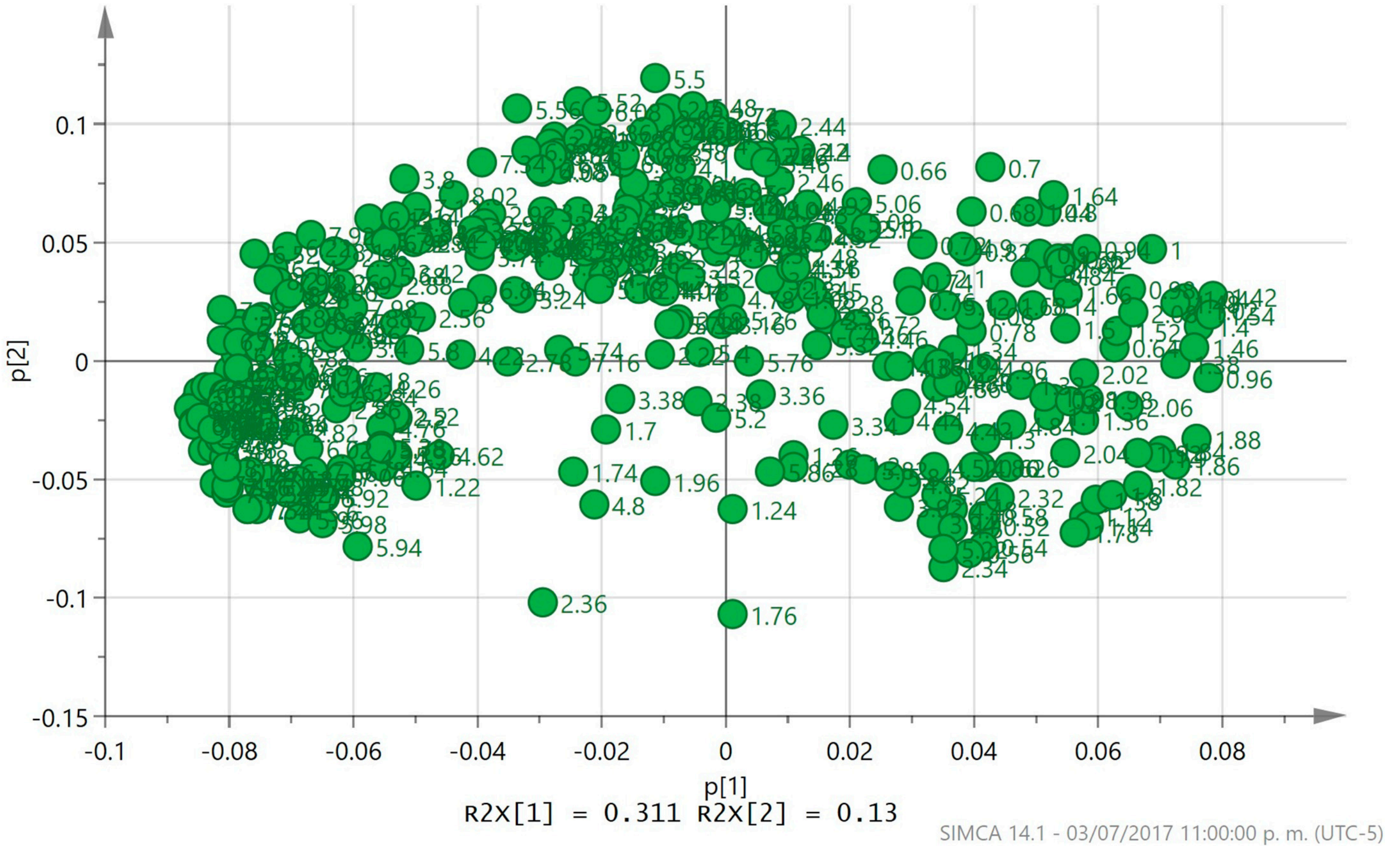

Figure S1. Scatter plot of the loadings on PC2 vs PC1 corresponding to the scores plot shown in Figure 2 of the main manuscript.

molecules

MDPI

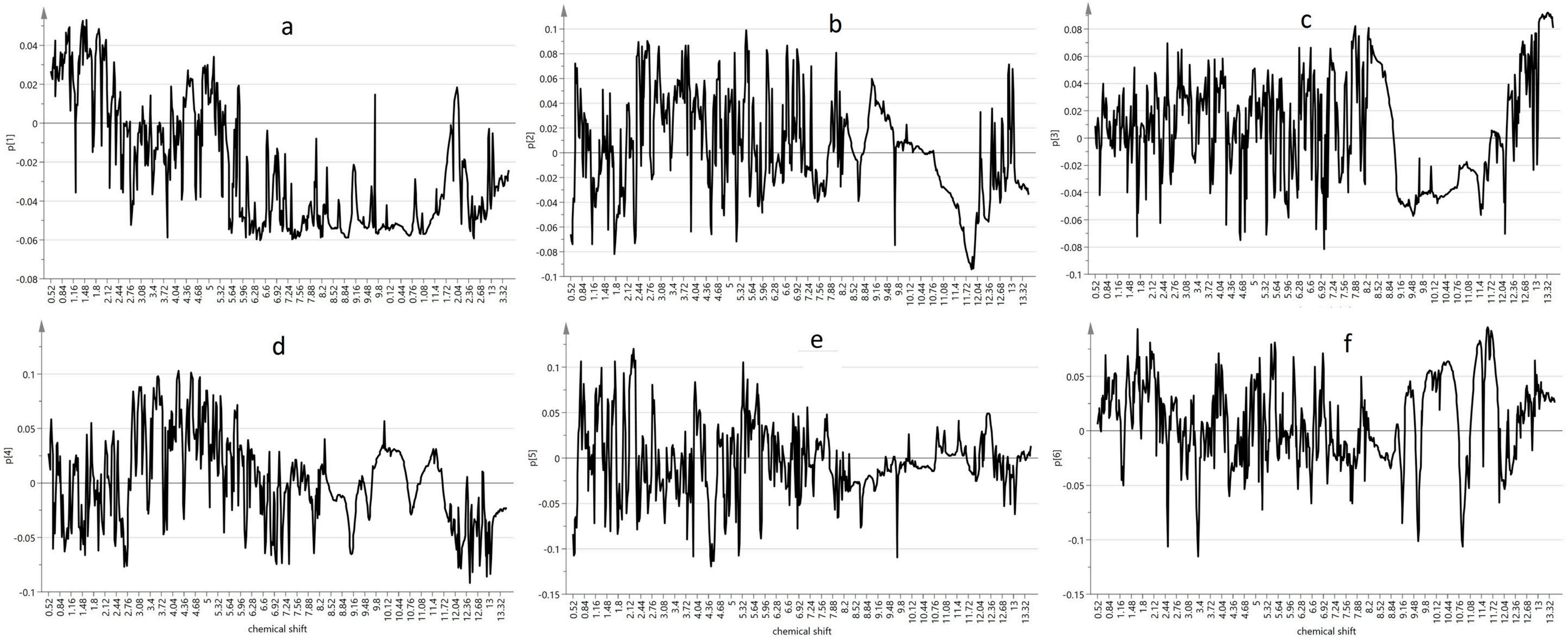

Figure S2. Loadings plots of the PCA analysis showing the a) first, b) second, c) third, d) fourth, e) fifth, and f) sixth principal component as a function of the NMR chemical shifts of the sample’s spectra.
